# Epidermal distribution of tetrodotoxin-rich cells in newly hatched larvae of *Takifugu* spp.

**DOI:** 10.1007/s10126-024-10377-x

**Published:** 2024-10-02

**Authors:** Keishiro Inahashi, Ryo Yonezawa, Kentaro Hayashi, Soichi Watanabe, Kazutoshi Yoshitake, Ashley Rinka Smith, Yui Kaneko, Inori Watanabe, Rei Suo, Shigeharu Kinoshita, Muhammad Ahya Rafiuddin, Yuki Seki, Arata Nagami, Hajime Matsubara, Nobuo Suzuki, Tomohiro Takatani, Osamu Arakawa, Miwa Suzuki, Shuichi Asakawa, Shiro Itoi

**Affiliations:** 1 Graduate School of Agricultural and Life Sciences, The University of Tokyo, Bunkyo, Tokyo 113-8657 Japan; 2 College of Bioresource Sciences, Nihon University, Fujisawa, Kanagawa 252-0880 Japan; 3 Noto Center for Fisheries Science and Technology, Kanazawa University, Ossaka, Noto-Cho, Ishikawa 927-0552 Japan; 4 Noto Marine Laboratory, Institute of Nature and Environmental Technology, Division of Marine Environmental Studies, Kanazawa University, Ogi, Noto-Cho, Ishikawa, 927-0553 Japan; 5 Graduate School of Integrated Science and Technology, Nagasaki University, Nagasaki, 852-8521 Japan; 6 Present Address: School of Marine Biosciences, Kitasato University, Sagamihara, Kanagawa 252-0373 Japan

**Keywords:** Epidermis, Fish larvae, Immunohistochemistry, Pufferfish, *Takifugu*, Tetrodotoxin (TTX)

## Abstract

Pufferfish of the genus *Takifugu* possess tetrodotoxin (TTX), known as “pufferfish toxin” and it is believed that pufferfish eggs and newly hatched larvae utilize TTX as a defensive substance against predators. However, the mechanism for the placement of TTX to specific cells on the larval body surface during the developmental process remains unknown. In this study, we clarify the distribution and characteristics of TTX-rich cells. We performed whole-mount immunohistochemistry (IHC) using anti-TTX monoclonal antibody on larvae of two pufferfish species, *Takifugu rubripes* and *Takifugu alboplumbeus*, just after hatching. This allowed observation of the TTX location and compared it with those of wheat germ agglutinin (WGA)-positive (periodic acid-Schiff (PAS)-positive) cells for mucous cells and IHC using anti-Na^+^/K^+^-ATPase (NKA) monoclonal antibody for ionocytes. As a result, uniformly scattered localization of TTX-rich cells was commonly observed in the epidermis of the larvae of the two *Takifugu* species. TTX-rich cells were WGA-negative (PAS-negative) and structurally distinct from NKA-positive cells, suggesting that TTX-rich cells are unreported small cells unique to pufferfish skin, but not mucous cells nor ionocytes.

**Supplementary Information:**

The online version contains supplementary material available at 10.1007/s10126-024-10377-x.

## Introduction

Pufferfish of the genus *Takifugu*, which are widely consumed in Japan under strict food hygiene management, possess tetrodotoxin (TTX), known as “pufferfish toxin” (Noguchi et al. 2006). Pufferfish are thought to accumulate TTX in their bodies through the food chain, including TTX-bearing animals, such as flatworm and ribbon worm, starting with bacteria as a primary producer (Noguchi and Arakawa 2008). In practice, grass puffer *Takifugu alboplumbeus* juveniles feed on TTX-bearing flatworms and incorporate composition of TTX and its analogs (Itoi et al. 2018b; Ueda et al. 2024). Tissue distribution patterns of TTX are variable among *Takifugu* species, with all species having at least some accumulations of TTX in the liver and ovary (Noguchi and Arakawa 2008). Some species accumulate TTX in the skin for predator protection (Kodama et al. 1985, 1986). In the toxicification process in adult tiger puffer *Takifugu rubripes*, TTX is mainly absorbed from the gastrointestinal tract into the vascular system (Matsumoto et al. 2008a, b). Subsequently, it is quickly transferred to the liver in an unbound or carrier-bound state (Matsumoto et al. 2007, 2008a, 2010). Part of TTX is transferred and accumulated in the skin of males and in the ovary of females via the liver in “torakusa”, a hybrid of tiger puffer *T. rubripes* and grass puffer *T. alboplumbeus* (Wang et al. 2011). Similarly, it has been confirmed that excess TTX in the liver is transferred to the epidermis of tiger puffer juveniles (Ikeda et al. 2009).

TTX in the ovary of *Takifugu* species will be localized on the larval body surface in association with embryogenesis after fertilization. It has been reported that some potential predatory fish respond to TTX in the skin of newly hatched pufferfish larvae and immediately spit them out, suggesting that it is clear that pufferfish larvae utilize TTX as a defensive substance against predatory fish (Itoi et al. 2014, 2018a). It was reported that adult grass puffer localizes TTX in sacciform cells, basal cells and mucous cells in the epidermis (Itoi et al. 2012), and tiger puffer localize exclusively in basal cells (Ikeda et al. 2009; Okita et al. 2013).

On the other hand, the density and the types of the TTX-rich cells on the body surface of pufferfish larvae remains unclear. Therefore, in this study, we focused on newly hatched puffer larvae, in which TTX-rich cells have been reported (Itoi et al. 2014, 2018a). Although conventional whole-mount staining could not be applied to thicker samples, we improved the technology for the tissue clearing treatment to suppress background fluorescence. Subsequently, whole-mount immunohistochemistry (IHC) was performed to observe TTX location throughout the epidermis in three dimensions. In addition, some methods of staining were performed to detect cell nuclei, mucous cells and ionocytes. These allowed us to elucidate the characteristics and distribution of TTX-rich cells on pufferfish larvae.

## Materials and Methods

### Reagents and Chemicals

TTX (purity ≥ 90%), paraformaldehyde (PFA) solution in phosphate buffer and clear, unobstructed brain/body imaging cocktails and computational analysis (CUBIC)-1 were purchased from FujiFilm Wako (Osaka, Japan). Anti-TTX monoclonal antibody was kindly provided from Dr. Kentaro Kawatsu (Osaka Prefectural Institute of Public Health, Osaka, Japan). VectaFluor Excel Amplified Anti-Mouse IgG, DyLight 594 Antibody Kit (containing normal horse serum, secondary antibody, and VectaFluor Reagent) and VECTASHIELD^®^ Vibrance™ Antifade Mounting Medium were obtained from Vector Laboratories, Inc. (CA, USA) and the anti-Na^+^/K^+^-ATPase (NKA) α-subunit (α5) antibody from the Developmental Studies Hybridoma Bank (IA, USA). The α5 antibody is a mouse monoclonal antibody against the avian NKA α-subunit and has been widely used to detect branchial NKA (Inokuchi et al. 2017). 4′,6-Diamidino-2-phenylindole, dihydrochloride (DAPI) solution was from Dojindo Laboratories (Kumamoto, Japan), wheat germ agglutinin (WGA), Alexa Fluor 488 Conjugate from Thermo Fisher Scientific (MA, USA) and Alcian Blue - PAS Stain Kit from ScyTek Laboratories (UT, USA).

### Pufferfish Larvae

Larvae of the tiger puffer *Takifugu rubripes* were obtained by artificial insemination of wild pufferfish parents that were captured by a set-net in the coastal waters off Noto, Ishikawa, Japan, conducted at Kanazawa University in April – May 2023. TTX (9.2 ± 1.1 μg/g) was detected in the ovaries from *T. rubripes* females which were used in the artificial insemination (Fig. S1). Larvae were fixed with 4% PFA solution in phosphate buffer (pH 7.4) on the day of hatching and stored at 4 °C until processing. Larvae of the grass puffer *Takifugu alboplumbeus* were obtained by artificial fertilization of wild pufferfish parents that were captured from the coastal waters off Kamogawa, Chiba, Japan, in June 2023. It has been reported that TTX is detected in the ovaries of all spawning *T. alboplumbeus* females at the sites where the pufferfish were collected for this study (Asano et al. 2023). The 0 day post-hatch (dph) larvae were fixed and stored in the same method as tiger puffer.

### Whole-Mount IHC

Samples were rinsed with phosphate buffered saline (PBS, pH 7.4), and immersed in a solution containing CUBIC-1/PBS (1:1, v/v) for 1 h, and then immersed in a CUBIC-1 solution overnight at room temperature (RT) as per the manufacturer’s protocol. Blocking was performed with 2.5% normal horse serum for 1 h at RT. IHC against TTX was performed using a 96-well cell culture plates (Thermo Fisher Scientific) with 2.0 µg/mL mouse anti-TTX monoclonal antibody (Kawatsu et al. 1997) overnight at RT. Samples were incubated at RT for 15 min with Amplifier Antibody, followed by 20 min at RT with VectaFluor Reagent. Samples were stained with 1 mg/mL DAPI solution (1:100) for 20 min followed by PBS wash. Samples were stained with 1.0 mg/mL WGA, Alexa Fluor 488 Conjugate (1:7) for 10 min. Some samples were stained as a negative control which was a sample with an absorbed antibody treated with a high concentration of TTX (10,000-fold in molar ratio to anti-TTX antibody) for 3 h at RT instead of anti-TTX antibody. The absorbed antibody was used after dilution with PBS without post-absorption purification.

To visualize ionocytes, mouse anti-NKA α-subunit antibody (1:500) was used in place of anti-TTX antibody, and its negative control was PBS in place of anti-NKA α-subunit antibody.

To compare the staining characteristics of mucous cells, WGA-stained samples were subjected to PAS staining using the Alcian Blue - PAS Stain Kit, excluding the Alcian Blue and Mayer’s Hematoxylin staining steps referred to the manufacturer’s protocol.

### Fluorescence Microscopy Analysis

Treated samples were sealed in VECTASHIELD^®^ Vibrance Antifade Mounting Medium on a glass slide. Observation of immunoreactivity image was done with an All-in-One Fluorescence Microscope BZ-X810 (Keyence, Osaka, Japan). The three dimensional (3D) images were acquired using the Z-stack and sectioning functions available with the microscope. Subsequently, the entire surface was two-dimensionally visualized using the stitching and full focus functions. Then, the level correction function was used to adjust the brightness levels.

## Results

**Fig. 1:**
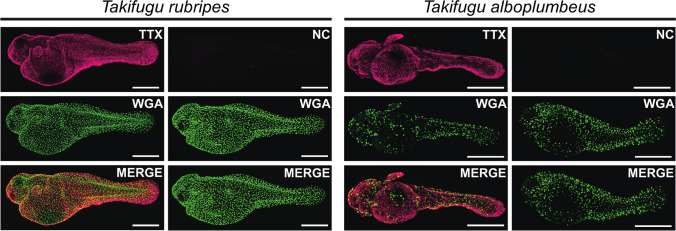
TTX location inferred from whole-mount IHC staining for 0-day post-hatch (dph) larvae of tiger puffer *Takifugu rubripes* and grass puffer *Takifugu alboplumbeus*. TTX, IHC with anti-TTX antibody (magenta); NC, negative control treated with anti-TTX antibody absorbed to TTX; WGA, fluorescence from WGA staining (green); MERGE, merged image with TTX (or NC) and WGA. Scale bars: 0.5 mm

**Fig. 2:**
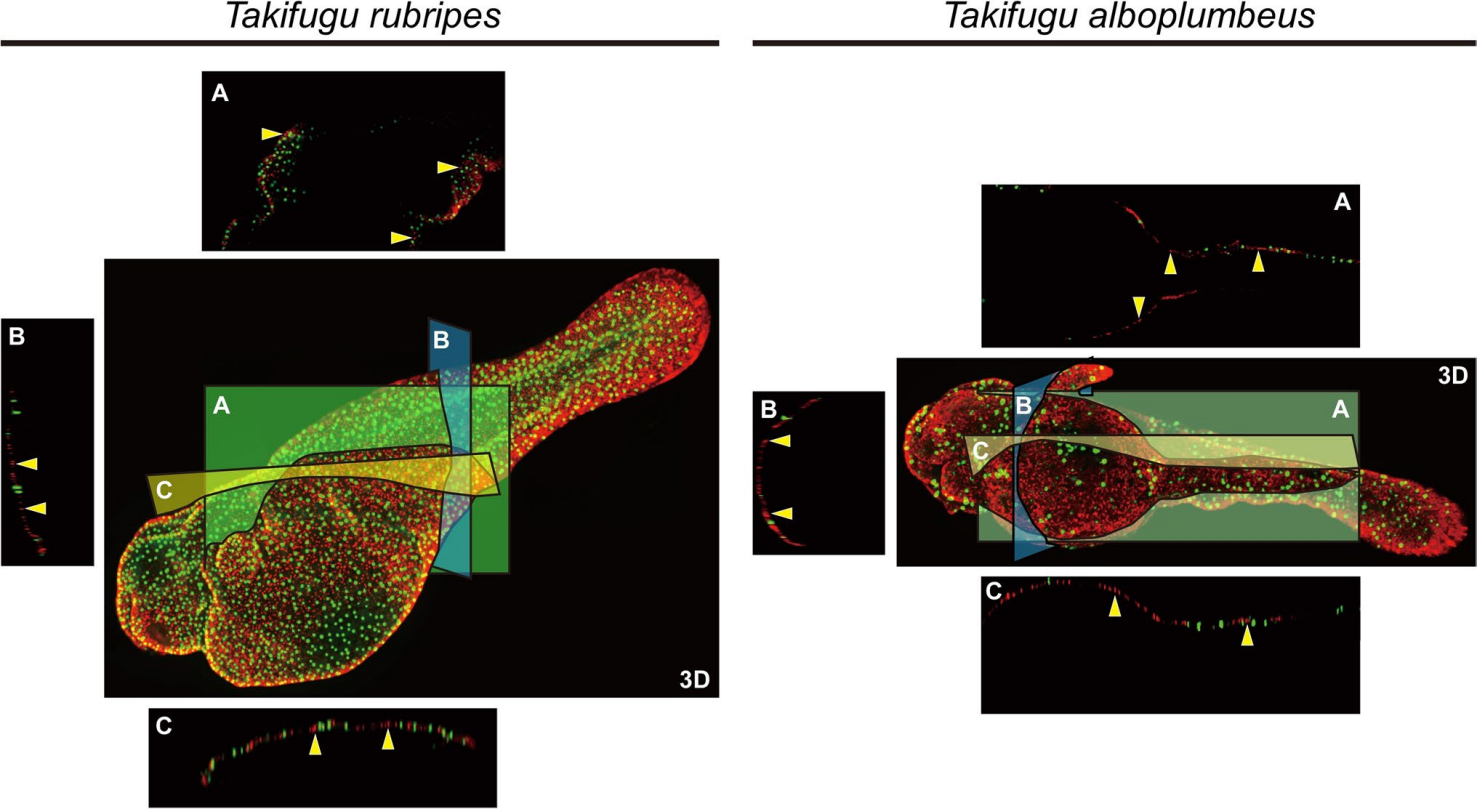
Three-dimensional (3D) arrangement of TTX-rich cells and WGA stained cells for larvae of tiger puffer *Takifugu rubripes* and grass puffer *Takifugu alboplumbeus*. Section like images (panels A–C) were extracted from 3D view. Signals for IHC with anti-TTX antibody are observed in magenta (yellow arrowheads), and fluorescence from WGA staining are in green

**Fig. 3:**
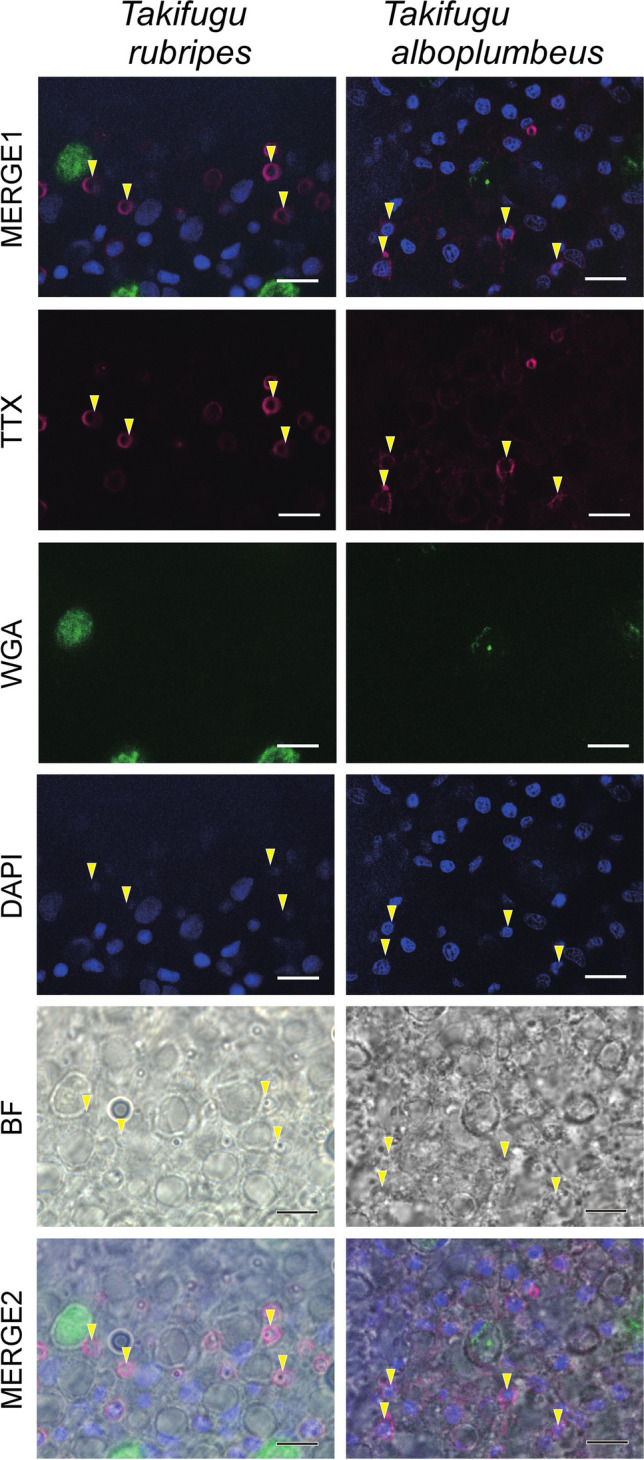
Details of TTX location in the tail epidermis of larvae (0 dph) of tiger puffer *Takifugu rubripes* and grass puffer *Takifugu alboplumbeus*. TTX, IHC with anti-TTX antibody (magenta); WGA, fluorescence from WGA staining (green); DAPI, DAPI staining (blue); BF, bright-field observation; MERGE1, merged image of panels of TTX, WGA and DAPI; MERGE2, merged image of panels of MERGE1 and BF. Yellow arrowheads represent positions for TTX-rich cells. Scale bars: 10 µm

Whole-mount IHC against TTX represented the specific signals in the whole epidermis of *T. rubripes* and *T. alboplumbeus* as the magenta spots (Fig. 1). As shown in Fig. 2, section-like observation using 3D display demonstrated that TTX signals were detected only in the epidermis. Intense fluorescence with anti-TTX antibody was detected in small cells, about 5 µm in diameter, located in the outermost layers of the epidermis (Fig. 3). No signal specific to TTX was observed in the central part of the cell, including the cell nucleus, which is stained with DAPI which binds even a very small amount of DNA and emits blue fluorescence.

Signals from WGA staining were scattered uniformly in the epidermis of the whole body except for the eyes (Fig. 1), and section-like observation using 3D display showed that WGA-positive cells were detected only in the epidermis (Fig. 2). Intense fluorescence with WGA staining was detected in the epidermal cells with a diameter of 10–20 µm (Fig. 3). WGA-positive cells were differently located from TTX-rich cells.

In thin tissues, such as the tail, cells stained with magenta by PAS staining (Fig. S2). On the other hand, in the head and abdomen, it was difficult to observe cells stained by PAS staining because of the tissue thickness. Therefore, we compared the same area stained by WGA and PAS staining in the tail. As a result, the cells with a diameter of approximately 10–20 µm that were stained magenta by PAS staining corresponded to the cells stained by WGA staining (Fig. S2).

**Fig. 4:**
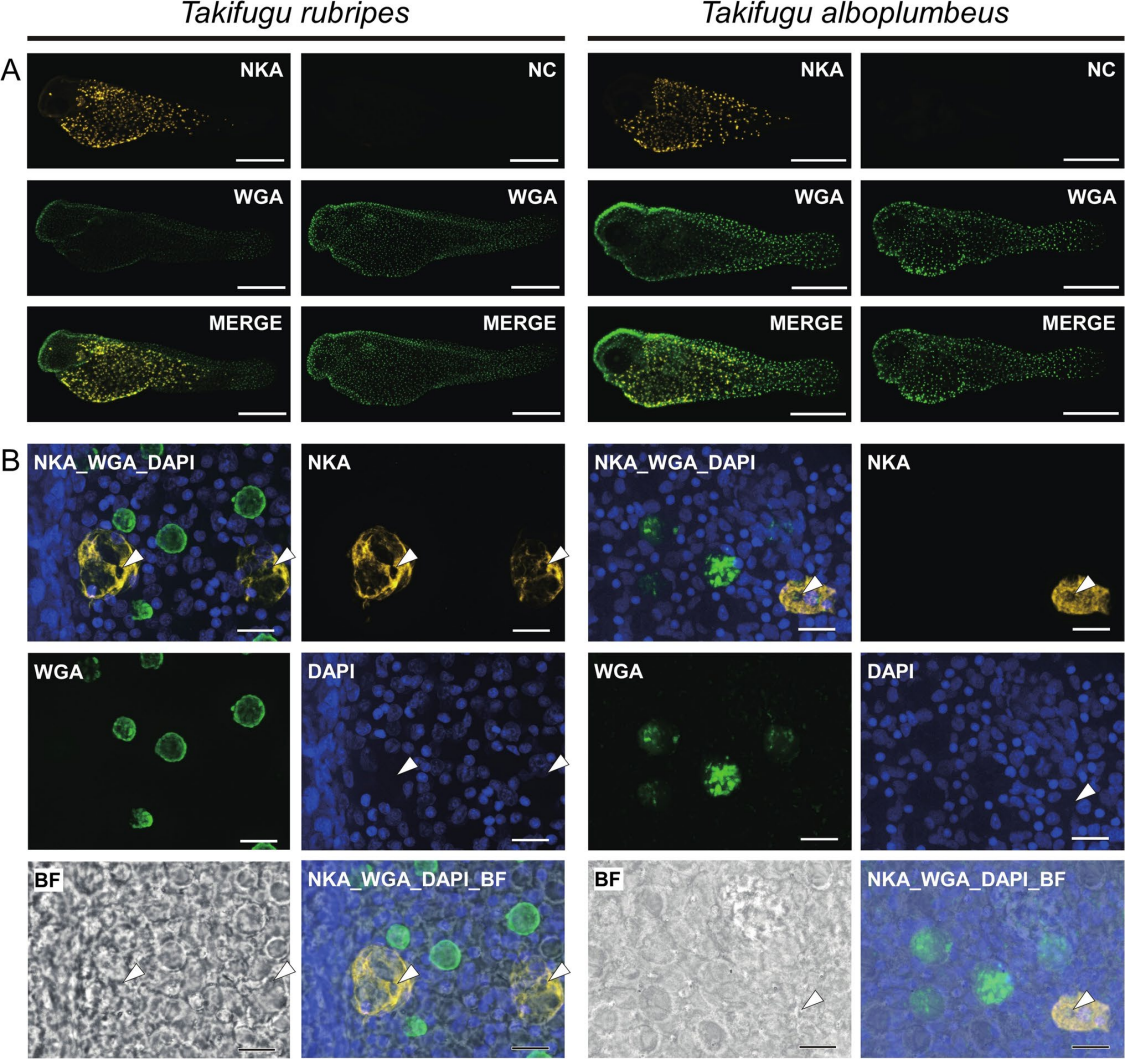
Ionocyte location in larvae (0 dph) of tiger puffer *Takifugu rubripes* and grass puffer *Takifugu alboplumbeus*. Panels in **A**: NKA, IHC with anti-NKA antibody (yellow); WGA, fluorescence from WGA staining (green); NC, negative control; MERGE, merged image with NKA (or NC) and WGA. Panels in **B**: NKA, whole-mount IHC with anti-NKA antibody (yellow); WGA, WGA staining (green); DAPI, DAPI staining (blue); BF, bright-field observation. Opening of the ionocyte has no positive reaction to DAPI (white arrowheads). Scale bars: panels in **A**, 0.5 mm; panels in **B**, 20 µm

Whole-mount IHC targeting NKA demonstrated that the specific signals of ionocytes concentrated around the yolk sac membrane with yellow spots (Fig. 4). Intense fluorescence with anti-NKA antibody was remarkable in the large epidermal cells with a diameter of about 30 µm. Opening of ionocytes is not located directly above the nucleus which is stained with DAPI (white arrowheads in Fig. 4).

## Discussion

Pufferfish larvae of the genus *Takifugu* possess maternally derived TTX on their body surface, and are thought to use it to avoid predation (Itoi et al. 2014, 2018a). However, it was not known which parts of the body surface of the pufferfish larvae were covered with TTX-rich cells, and the details of the cells that retained TTX were not clear.

IHC with anti-TTX antibodies in the skin of *Takifugu* species has shown that TTX-rich cells are contained in sacciform cells, mucous cells, basal cells of adult grass puffer (Itoi et al. 2012), basal cells of juvenile tiger puffer (Okita et al. 2013), glands of the pear puffer *Takifugu vermicularis* (Mahmud et al. 2003) and gland-like structures of adult fine-patterned puffer *Takifugu flavipterus* (Sato et al. 2021). Whole-mount IHC in this study revealed that TTX-rich cells cover the entire body, except the eyes, and that these cells are small and distinct from mucous cells and ionocytes, suggesting that these cells are pufferfish-specific or TTX-bearing fish-specific. The possibility that TTX-rich cells correspond to sacciform cells of adult pufferfish was also ruled out, as the results showed that TTX-rich cells were significantly smaller than mucous cells, which in turn are smaller than sacciform cells of adult pufferfish (Itoi et al. 2012; Tsutsui et al. 2018). This suggests that TTX-rich cells in *Takifugu* larvae cannot be identified based on the information from previous studies of pufferfish.

Adult *Acanthopterygii* species, including pufferfish, generally have epithelial cells, mucous cells and sacciform cells in the epidermis, and the epidermis and dermis are separated by a basal cell layer (Takashima and Hibiya 1995). In fish larvae, sacciform cells are not observed, with exceptions in flatfish (Sarasquete et al. 1998; Padrós et al. 2011). Epidermis of newly hatched larvae of *Acanthopterygii* species has been observed in marine flatfish (Roberts et al. 1973; Sarasquete et al. 1998; McFadzen et al. 2000; Campinho et al. 2007; Padrós et al. 2011), and fresh or brackish water fish, tilapia (Shiraishi et al. 1997; Hiroi et al. 1999, 2005, 2008; Uchida et al. 2000; Kaneko and Shiraishi 2001). The epidermis of newly hatched larvae is generally composed of 1 or 2 cell layers, with epithelial (squamous) cells, mucous cells, mucous-free cells which have the same morphology as mucous cells (mucous cell-like cells), and ionocytes (chloride cells; mitochondria-rich cells), and is separated from the dermis by a basal cell layer. Views on mucous cell-like cells vary as sacciform/mucous cells (Sarasquete et al. 1998) and saccular/sacciform cells (Padrós et al. 2011), and these secretory cells are the same size or larger than mucous cells. On the other hand, the TTX-rich cells observed on the body surface of pufferfish larvae were remarkably smaller and more overcrowded than mucous cells, suggesting that they differ from the known secretory cells described above. The ionocytes are known to be large cells although there have been no observations of these in pufferfish larvae (Hiroi et al. 2008; Inokuchi et al. 2022). The ionocyte’s opening of pufferfish larvae appeared to be the same size (5–10 µm) as the TTX-rich cells which are observed in this study, and the nucleus was not observed directly below the opening of ionocytes. This is inconsistent with TTX-rich cells having a nucleus. These results suggest that TTX-rich cells of *Takifugu* larvae are different from any cells previously targeted for observation in larval fish skin. TTX-rich cells are expected to exocrine secretion of TTX against predators although at this time no details of the openings were observed and there is no evidence of exocrine secretion. Further studies are required.

Unknown TTX-binding substance may be involved in TTX localization of pufferfish larvae, although the process of TTX localization in the skin of them has not been reported. The IHC signals specific to TTX observed in this study suggest that TTX is in a protein-bound form to be retained in the tissue because free-TTX would have been washed out during the fixation process as shown by Yonezawa et al. (2024). In practice, the cavity in the gland and the gland-like structure of adult pufferfish does not exhibit staining in IHC with anti-TTX antibody (Mahmud et al. 2003; Sato et al. 2021). In addition, TTX-rich cells in this study were WGA- and PAS-negative. PSTBP, which is known as a carrier protein of TTX, is a PAS-positive glycoprotein (Yotsu-Yamashita et al. 2001) and it is suggested that the major protein in the WGA-bound fraction is an isoform derived from a PSTBP-like gene (Zhang et al. 2023). These indicate that TTX observed in this study may bind to a different substance from the PSTBP-like protein. Further investigation along this line would be necessary.

In conclusion, our study attempted to collect basic knowledge to clarify the localization mechanism of maternal TTX in the skin of the pufferfish larvae. TTX-rich cells in pufferfish larvae were found only on the body surface and not corresponding with basal cells, mucous cells and ionocytes. Our data inferred that TTX-rich cells observed in pufferfish larvae are not classified into ionocytes, and known secretory cells, such as mucous cells, sacciform cells and saccular cells. In the future, we aim to identify TTX-rich cell types using electron microscopy analysis and single-cell transcriptome analysis. This will provide insights into the accumulation of TTX, the presentation mechanisms of TTX in pufferfish larvae and the retention/localization of TTX in tissues across their life stages.

## Supplementary Information

Below is the link to the electronic supplementary material.

Supplementary file1 (PDF 4829 KB)

## Data Availability

Data will be made available on request.
