# Supplementary material for: Epidermal distribution of tetrodotoxin-rich cells in newly hatched larvae of Takifugu spp
Source: Mar Biotechnol (NY). 2024 Oct 2;26(6):1367–74. doi: 10.1007/s10126-024-10377-x (PMC11541287; doi:10.1007/s10126-024-10377-x)
Supplement: Supplementary file 1 — Supplementary file1 (PDF 4829 KB) [file 10126_2024_10377_MOESM1_ESM.pdf]

Epidermal distribution of tetrodotoxin-rich cells in newly-hatched larvae of *Takifugu* spp.

Keishiro Inahashi[a], Ryo Yonezawa[a], Kentaro Hayashi[a], Soichi Watanabe[a], Kazutoshi Yoshitake[a,†], Ashley Rinka Smith[a], Yui Kaneko[b], Inori Watanabe[b], Rei Suo[b], Shigeharu Kinoshita[a], Muhammad Ahya Rafiuddin[c], Yuki Seki[c], Arata Nagami[c], Hajime Matsubara[c], Nobuo Suzuki[d], Tomohiro Takatani[e], Osamu Arakawa[e], Miwa Suzuki[b], Shuichi Asakawa[a,]*, Shiro Itoi[b,]*

[a] Graduate School of Agricultural and Life Sciences, The University of Tokyo, Bunkyo, Tokyo, 113-8657, Japan

[b] College of Bioresource Sciences, Nihon University, Fujisawa, Kanagawa, 252-0880, Japan

[c] Noto Center for Fisheries Science and Technology, Kanazawa University, Ossaka, Noto-Cho, Ishikawa, 927-0552, Japan

[d] Noto Marine Laboratory, Institute of Nature and Environmental Technology, Division of Marine Environmental Studies, Kanazawa University, Ogi, Noto-Cho, Ishikawa, 927-0553, Japan

[e] Graduate School of Integrated Science and Technology, Nagasaki University, Nagasaki, 852-8521, Japan

* To whom correspondence should be addressed. E-mail: asakawa@g.ecc.u-tokyo.ac.jp (SA), sitoi@nihon-u.ac.jp (SI)

[†] Present address: School of Marine Biosciences, Kitasato University, Sagamihara, Kanagawa, 252-0373, Japan

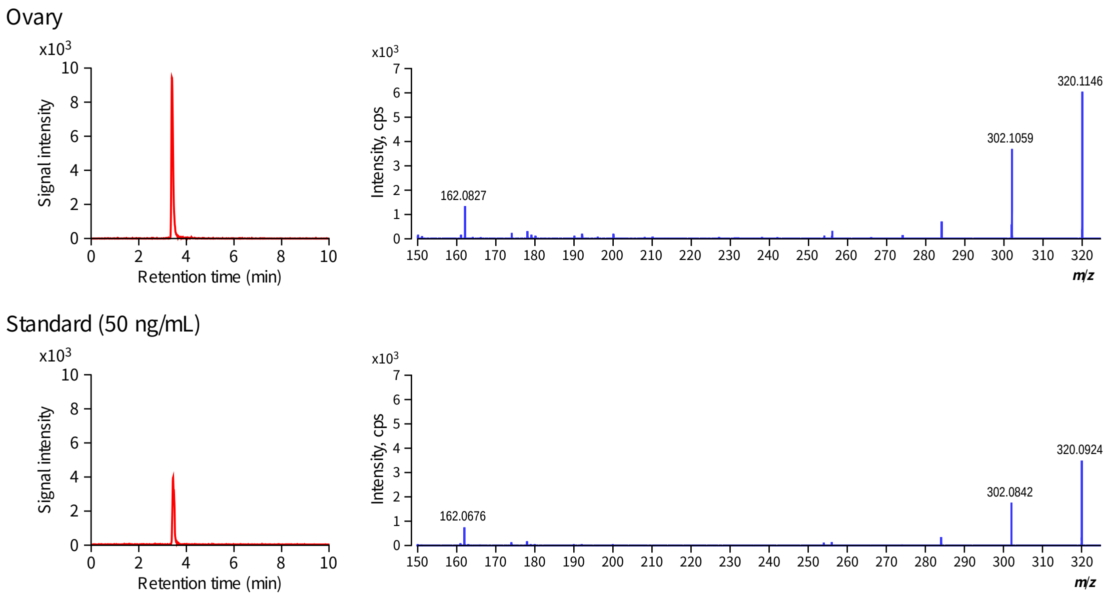

Fig. S1. Typical LC-MS/MS patterns of TTX from ovary of the pufferfish *Takifugu rubripes*, and 50 ng/mL standard (FUJIFILM Wako Pure Chemicals). Left and right panels represent LC-MS/MS chromatograms and the precursor/product ion mass spectra for TTX, respectively. TTX was extracted and purified in accordance with Miyazaki et al. (2024). LC-MS/MS was carried out on a Shimadzu LC-20AD solvent delivery system connecting to a SCIEX X500R Q-TOF mass spectrometer with an ESI source, according to Ito et al. (2023). The standard calibration curve was created using 1-100 ng/mL of standards which showed good linearity and precision (y = 392.75x + 350.46, R^2 = 0.99).

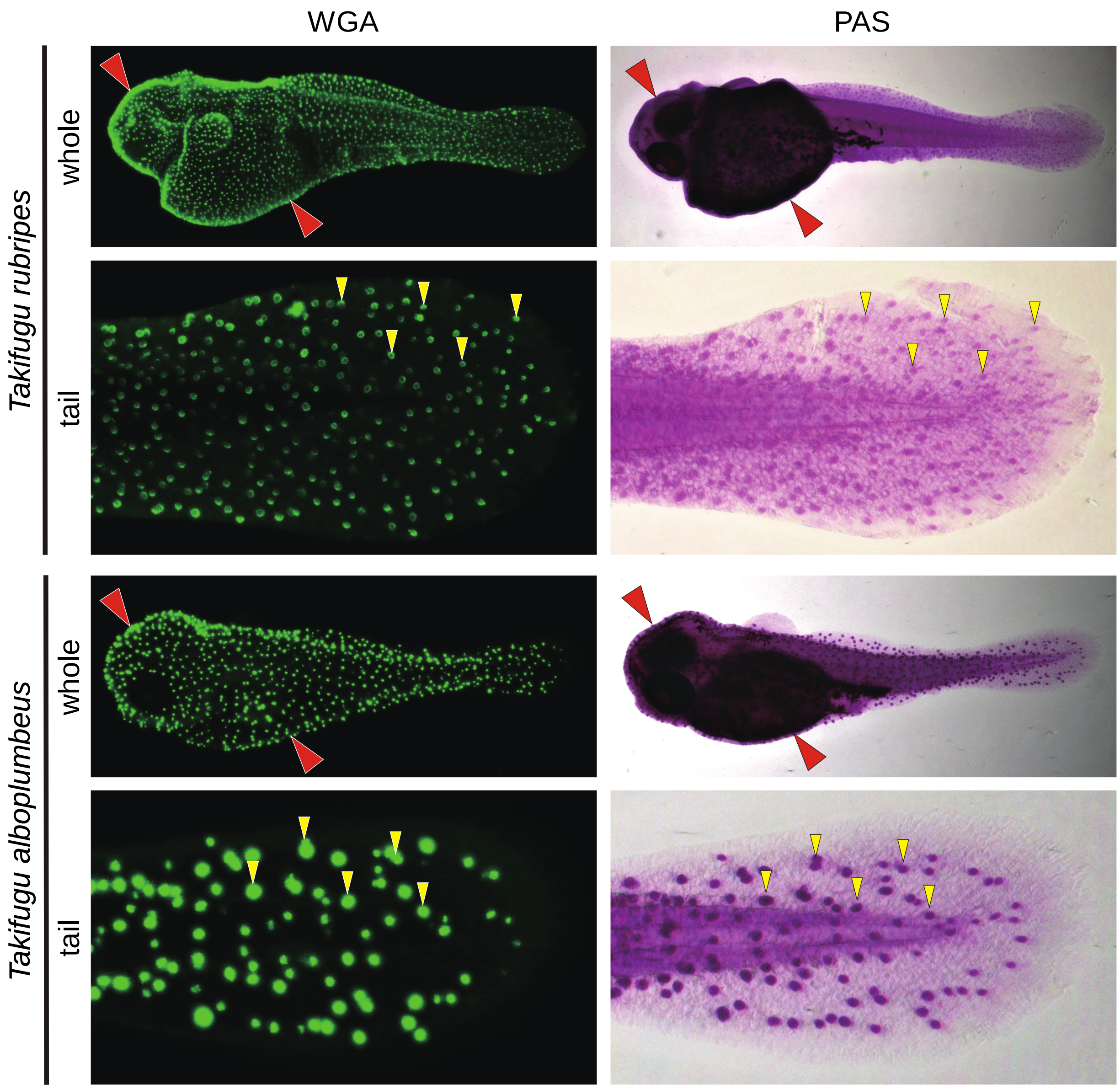

Fig. S2. Co-staining of WGA and PAS staining images in larvae (0 dph) of tiger puffer *Takifugu rubripes* and grass puffer *Takifugu alboplumbeus*. WGA, WGA staining (green); PAS, PAS staining of the same individuals (magenta). Unlike PAS staining, WGA staining allows the observation of positive cells even in the head and abdomen, where the tissue is thicker (red arrowheads). Most of the WGA-positive cells matched with PAS-positive cells (yellow arrowheads).

References

Ito M, Shirai K, Oyama H, Yasukawa S, Asano M, Kihara M, Suo R, Nakahigashi R, Adachi M, Nishikawa T, Itoi S (2023) Geographical differences in the composition of tetrodotoxin and 5,6,11-trideoxytetrodotoxin in Japanese pufferfishes and their origins. Chemosphere 336: 139214

Miyazaki K, Suo R, Itoi S, Hirota J, Adachi M, Miyasaka T, Nishikawa T, Yokoyama T, Sato S, Takada K (2024) 5, 6, 11-trideoxy tetrodotoxin attracts tiger puffer *Takifugu rubripes*. Toxicon 237: 107539
